# Fetal growth restriction: Case definition & guidelines for data collection, analysis, and presentation of immunization safety data

**DOI:** 10.1016/j.vaccine.2017.01.042

**Published:** 2017-12-04

**Authors:** Sarah Rae Easter, Linda O. Eckert, Nansi Boghossian, Rebecca Spencer, Eugene Oteng-Ntim, Christos Ioannou, Manasi Patwardhan, Margo S. Harrison, Asma Khalil, Michael Gravett, Robert Goldenberg, Alastair McKelvey, Manish Gupta, Vitali Pool, Stephen C. Robson, Jyoti Joshi, Sonali Kochhar, Tom McElrath, The Brighton Collaboration Fetal Growth Restriction Working Group

**Affiliations:** a Division of Maternal-Fetal Medicine, Brigham and Women’s Hospital, Harvard Medical School, Boston, MA, USA; b Department of Obstetrics and Gynecology, University of Washington, Seattle, WA, USA; c Department of Epidemiology & Biostatistics, Arnold School of Public Health, University of South Carolina, Columbia, SC, USA; d Consultant in Obstetrics, Institute for Women’s Health, University College London, UK; e Consultant Obstetrician, St Thomas' Hospital, London, UK; f Consultant in Obstetrics and Fetal Medicine, John Radcliffe Hospital, University of Oxford, Oxford, UK; g Division of Maternal-Fetal Medicine, Wayne State University, Detroit, MI, USA; h Department of Obstetrics and Gynecology, Columbia University Medical Center, New York, NY, USA; i Consultant in Obstetrics and Subspecialist in Fetal Medicine, St George’s University of London, London, UK; j Consultant in Obstetrics and Fetal Medicine, Norfolk and Norwich University Hospital, Norwich, UK; k Consultant Obstetrician, Subspecialist in Maternal and Fetal Medicine, Barts Health NHS Trust, London, UK; l Director of Scientific and Medical Affairs, Sanofi Pasteur, Swiftwater, PA, USA; m Professor of Fetal Medicine, Newcastle University, Newcastle upon Tyne, UK; n Deputy Director of Immunization Technical Support Unit, Public Health Fund of India, New Delhi, India; o Global Healthcare Consulting, New Delhi, India; p Erasmus University Medical Center, Rotterdam, The Netherlands

## 1 Preamble

### 1.1 Need for developing case definitions and guidelines for data collection, analysis, and presentation for fetal growth restriction as an adverse event following immunization

Fetuses that fail to meet their growth potential in utero are at risk for adverse antenatal and postnatal events such as stillbirth, preterm birth, and adverse neonatal and long-term health outcomes [1], [2], [3], [4], [5]. Therefore, antenatal recognition and monitoring of fetal growth restriction (FGR) is an important component of prenatal care [6], [7], [8]. Despite the clinical and public health importance of this problem there is no universally accepted definition of FGR [9], [10]. Furthermore, terminology such as intrauterine growth restriction (IUGR) or small for gestational age (SGA) are used interchangeably and without specificity to describe this clinical entity. In its simplest form, FGR is defined as a sonographic estimation of fetal weight below the tenth percentile for a given gestational age [11], [12], [13], [14]. Though this definition is simple to understand and translating into practice, it is an inadequate definition for FGR.

FGR can be a consequence of maternal, fetal, or placental factors. Diagnosing all fetuses with an estimated fetal weight (EFW) below the tenth percentile with FGR fails to account for the individual growth potential of each fetus. Constitutionally small fetuses who might be expected to have a lower birthweight based on parental characteristics may be misdiagnosed as pathologically small [15]. Conversely, fetuses destined for a higher birthweight may fail to reach their growth potential due to a pathologic process yet never fall below a threshold based on fetal or birth weight below a specific centile (e.g. 10th) [16]. An ideal definition of FGR would detect those fetuses with a pathologic failure to meet their growth potential subsequently at risk of adverse outcomes.

Numerous studies have attempted to improve the sensitivity and specificity of the definition through adjunct testing and optimization of growth curves used to define the tenth percentile diagnostic cutoff. The sentinel investigations into FGR used measurements of the fetal head, abdomen, and femur to develop growth curves within small homogenous patient populations [17]. Though these measurements yielded reliable estimations of fetal weight, the growth curves lacked generalizability, particularly in an international context [18]. Contemporary studies on FGR have advocated individualized growth curves accounting for maternal and fetal characteristics such as ethnicity and gender to solve this dilemma [19], [20], [21]. However, large-scale international prospective studies of healthy pregnancies show little difference in growth curves between populations [22]. Additional studies investigating the utility of adjunct studies such as amniotic fluid assessment and use of Doppler attempt to further clarify the definition of FGR [23], [24].

Despite these controversies in defining FGR, its detection is an important component of antenatal care. The majority of the prior vaccine studies in pregnant women, including specifically those focused on obstetric outcomes, do not address FGR as an adverse outcome [25], [26], [27], [28]. Some authors have reported neonatal outcomes including identification of low birth weight (LBW) and SGA infants without an attempt to detect these events in pregnancy [29], [30], [31]. Though neonatal disorders of growth potential could be considered a postnatal diagnosis of FGR, they are different diagnoses with distinct implications within the context of studies on immunizations.

The likely cause of pathologic FGR can vary in according to clinical setting. Some etiologies of FGR, such as preeclampsia or congenital anomalies, may be similar across clinical settings. FGR associated with maternal comorbidity such as advanced maternal age or gastric bypass surgery can be considered unique to countries with higher healthcare related expenditures [32], [33]. In contrast FGR in lower income countries is more likely to be associated with malnutrition or parasitic diseases, with malaria being the classic example [34], [35], [36], [37], [38].

This relationship between maternal infection and FGR is well described for many diseases—even in the absence of congenital infection [39], [40], [41], [42], [43], [44]. Specifically, FGR has been described as a consequence of vaccine-preventable illnesses, such as influenza [45], [46]. As maternal vaccination becomes an increasingly prioritized component of routine prenatal care, monitoring for adverse vaccine-related outcomes gains similar importance. The complex interplay between FGR, infection, and medical comorbidity makes early detection and diagnosis of this pregnancy complication of paramount importance. Timely diagnosis of a pathologic disorder of growth potential in utero, as opposed to relying solely on a postnatal diagnosis of a pathologically small infant, is necessary to identify a temporal relationship between the diagnosis of FGR and a vaccine of interest.

There is a paucity of data on FGR in existing vaccine trials, perhaps in part due to the controversy surrounding the diagnosis within the medical community. Given the clinical variation in the definition, the absence of a uniformly accepted definition of FGR following immunizations is not surprising. This is, however, a missed opportunity, as data comparability across trials or surveillance systems would facilitate data interpretation and promote the scientific understanding of the event.

### 1.2 Methods for the development of the case definition and guidelines for data collection, analysis, and presentation for fetal growth restriction as an adverse events following immunization

Following the process described in the overview paper as well as on the Brighton Collaboration Website http://www.brightoncollaboration.org/internet/en/index/process.html, the Brighton Collaboration *Fetal Growth Restriction Working Group* was formed in 2015 and included members from clinical, academic, public health, and industry backgrounds [47]. The composition of the working and reference group as well as results of the web-based survey completed by the reference group with subsequent discussions in the working group can be viewed at: http://www.brightoncollaboration.org/internet/en/index/working_groups.html.

To guide the decision-making for the case definition and guidelines, a literature search was performed using Medline, Embase and the Cochrane Libraries, including the terms [fetal (Fetal) growth restriction or retardation], [intrauterine growth restriction or retardation] and [small for gestational age]. The search resulted in the identification of 23,441 English-language references, 5480 of which were published within the past five years. All abstracts were screened for relevance to a contemporary definition of FGR in a singleton pregnancy with particular attention to those related to infection, immunization, and under-represented countries. 102 articles with potentially relevant material were reviewed in more detail, in order to identify studies using case definitions or, in their absence, providing clinical descriptions of the case material. The literature search revealed extensive literature on the definition of FGR and development of associated growth curves and adjunct testing. No immuzation-related studies contained definitions of FGR and this outcome was seldom discussed. The most commonly encountered definitions were in medical society statements and contained substantial variation in both terminology and definitions. Similar heterogeneity was found in the definition of FGR throughout scientific studies addressing outcomes and management of this pregnancy complication. An inventory comprising the 102 relevant articles along with society definitions of FGR was made available to working group members via the Collections feature of MyNCBI.

### 1.3 Rationale for selected decisions about the case definition of fetal growth restriction as an adverse event following immunization

#### 1.3.1 The term fetal growth restriction

Terms such as intrauterine growth restriction (IUGR) and small for gestational age (SGA) are often used in clinical practice interchangeably with FGR. The term SGA has been proposed by some groups, including the Brighton Collaborative, as a diagnosis limited to neonates [11], [48]. Other society guidelines suggest using IUGR to identify those fetuses at risk of pathologic growth restriction and limiting the use of SGA to reference a constitutionally small fetus without evidence of pathology [12], [13], [14], [49]. In order to distinguish between a neonatal and fetal diagnosis of disorders of growth, use of the term SGA to reference a fetal disorder of growth will be avoided. IUGR and FGR are used interchangeably with less confusion as both clearly reference a diagnosis of growth restriction established prior to delivery. To limit confusion between these variably defined terms the Brighton definitions will utilize the term fetal growth restriction to define this adverse advent with levels of diagnostic certainty to further describe concern for pathologic FGR.

#### 1.3.2 Formulating a case definition that reflects diagnostic certainty: weighing specificity versus sensitivity

The number of sonographic findings that will be documented for each case may vary considerably depending on availability of technology in a given setting and availability of additional clinical information, such as pregnancy dating, critical to establishing a diagnosis. The case definition has been formulated such that the Level 1 definition is highly specific for the condition. As maximum specificity normally implies a loss of sensitivity, an additional diagnostic level has been included in the definition, offering a stepwise increase of sensitivity from Level 1 to Level 2, while retaining an acceptable level of specificity at all levels. Each Level has been further subdivided into subcategories of A and B in an attempt to better define pathologic FGR. Within both Levels, a subgroup of A provides better specificity and certainty for a pathologic process. Level B may be more sensitive for FGR but includes less specific findings with less certainty for its pathology. In this way it is hoped that all possible cases of FGR can be captured with clarity as to the concern for a disorder of fetal growth potential.

It needs to be emphasized that the grading of definition levels is entirely about diagnostic certainty, not clinical severity of an event. Thus, a clinically very severe event may appropriately be classified as Level Two rather than Level One if it could reasonably be of non-FGR etiology (e.g. in cases of limited evidence of pregnancy dating). Detailed information about the severity of the event should additionally always be recorded, as specified by the data collection guidelines.

#### 1.3.3 Rationale for individual criteria or decision made related to the case definition

FGR is most often diagnosed by use of ultrasound. Magnetic resonance imaging (MRI) of the fetus and placenta shows potential for furthering understanding of pathophysiology. However, this technology is time-consuming, expensive, and primarily restricted to research settings limiting its use for inclusion in the Brighton definition of FGR. At the other end of the spectrum, exam findings such as symphysis fundal height measurements are clinically ubiquitous for the detection of FGR [50]. This clinical measurement lacks sensitivity, offers little insight into potential for pathology, and lacks evidence for routine use limiting its utility in a research setting [51]. Additional studies such as placental pathologic examination, placental biomarkers, and related clinical diagnoses may offer insight into the underlying pathophysiology for FGR [52], [53]. This adjunct testing offers clinical utility for screening for and further understanding of FGR but lends little to establishing the initial diagnosis. Therefore, the Brighton case definition of FGR will focus on use of a combination of B-mode and Doppler ultrasound technology to establish the diagnosis of FGR. This approach should allow for adequate sensitivity and specificity without making identification of the adverse event overly cumbersome for investigators in study settings with limited resources. Ultrasound may not be universally available in all clinical settings, but its presence is requisite for diagnosis of FGR as an adverse event within the context of vaccine trials. In settings where ultrasound is unavailable for an antenatal diagnosis of disorders of fetal growth, investigators may use the Brighton Definition for Small for Gestational Age neonates as an alternative [48].

The most basic definition of FGR includes a fetus with a sonographic estimation of fetal weight below the tenth percentile for a given gestational age with increasing specificity for adverse perinatal outcomes below the third percentile [24]. A variety of fetal biometric measurements and functional Doppler studies have been studied to optimize the detection of FGR. Biometric parameters typically involve an assessment of head size (e.g. biparietal diameter (BPD), head circumference (HC), occipitofrontal diameter (OFD)), abdominal size (e.g. abdominal circumference (AC) or abdominal diameter (AD)), and femur length [54], [55]. A comprehensive guide to the technical performance of these measurements and other sonographic technique is beyond the scope of this paper, but several key points must be emphasized. Healthcare providers performing obstetric ultrasound should be trained in proper technique with assessment of quality to ensure inter- and intra-rater reliability. Selected sonographic studies should follow international published standards for obtaining and reporting measurements with appropriate references to resources used during scientific publication.

As previously described, the choice of a specific growth curve and its associated characteristics is a subject of ongoing debate in the obstetric community based on wide variation between published reference curves [18]. Many publically available growth curves currently available in ultrasound machines were derived from small homogenous populations [17]. Customized growth curves incorporating maternal and fetal characteristics have shown improved specificity over population-based curves in individual studies yet fail to demonstrate superiority in the most recent meta-analyses [19], [21], [56]. Contemporary population-based growth curves derived from a diverse population of low-risk pregnancies such as those from the INTERGROWTH-21st package show little variation in fetal size across non-isolated populations [22], [57], [58]. Yet translation of these growth standards into clinical practice is ongoing with presumably limited access to this data in existing technology [59]. The majority of existing society guidelines either lack recommendations on specific growth curve use or suggest employing a customized curve [12], [14], [49]. However, all of these guidelines were developed before the recent publication of newer fetal growth standards and expert opinion varies [10], [60], [61].

Resolving the ongoing debate of the ideal growth curve selection is beyond the scope of this project. Furthermore, prescribing a specific standard may unduly restrict participation of study sites with limited resources to access additional growth curves. Though the Brighton definition of FGR does not include a single specific growth curve, several key features of a selected growth curve should be emphasized. Study sites in any given trial should select a single curve to allow for meaningful comparison between sites. Selected growth curves should be internationally validated using a large sample size and published as a peer-reviewed study with an available reference. An optimal curve would be based on pregnancies with reliable early pregnancy dating, prospective data collection, and validation of measurements to avoid intra-observer bias.

In addition to a sonographic estimation of fetal weight, additional techniques can be used to aid in the diagnosis of FGR. As with the acquisition of measurements, adherence to existing performance standards should be ensured when performing this adjunctive testing [62]. These additional studies can include an assessment of amniotic fluid volume and Doppler velocities through varying maternal, placental, and fetal vessels where technology allows. Amongst the variety of Doppler studies available, assessment of velocities in the umbilical artery is one of the most accessible both in terms of technical ease and interpretation. Absence or reversal of diastolic velocities in the umbilical artery has been associated with adverse perinatal outcomes that are rare in the setting of normal umbilical artery velocities [63]. Inclusion of umbilical artery Doppler waveforms in the definition allows for added certainty in the diagnosis of pathologic growth restriction with minimal additional time or skill.

Doppler studies of the middle cerebral artery (MCA), ductus venosus, uterine arteries and the relationships between these values (e.g. the cerebral placental ratio (CPR)) have also been employed in the clinical description and prediction of FGR. Contemporary expert consensus guidelines attempting to better characterize early-onset and late-onset FGR underscore the clinical utility of measurements such as the CPR and the umbilical artery pulsatility index (PI) in assessment of FGR [60]. The PI is calculated using the peak systolic velocity, end diastolic velocity, and time averaged velocity in a given artery and comparing this value to existing standards for a given gestational age. An umbilical artery PI above the 95%ile has been associated with adverse perinatal outcomes similar to absent or reversed diastolic frequencies but with lower incidence [24]. This clinically useful measurement would likely improve the specificity of pathologic growth restriction, but has a slightly greater technical requirement with a need for further interpretation using a normative standard. The working group balanced specificity for FGR with establishing a case definition that can be achieved across a wide range of clinical settings with varying access to equipment and operator training. For this reason, pulsatility indices were not included in the case definition of FGR.

Similar reasoning guided the exclusion of CPR in the group’s definition of FGR. The CPR is derived by comparing the PI in the umbilical artery to that in the MCA and has a similar role in predicting adverse outcomes likely due to its role in detecting brain-sparing growth restriction [64], [65]. Brain-sparing growth restriction in which a fetus redirects blood flow away from the viscera in favor of the brain is associated with poorer perinatal outcomes [66]. Aside from an abnormal CPR, this subset of FGR is suggested by an abdominal circumference lagging the estimated weight of the fetus [24]. An isolated abdominal circumference below the tenth percentile can be associated with adverse outcomes and has been utilized in recent consensus guidelines to define pathology [24], [60]. Reference standards for this individual measurement have similar variation and challenges to those involving estimated fetal weight and rely on a single measurement to define pathology [18]. Though assessment for brain-sparing using these techniques would add specificity to the pathologic FGR diagnosis, it would involve significant additional technical requirements as well as increase the risk of false positives due to technical error. Based on this information and the absence of recommendation for routine use in existing clinical guidelines use of Doppler other than velocimetry in the umbilical artery are not included in the case definition for FGR [23], [24].

Assessment of amniotic fluid volume is a routine component of prenatal ultrasound and diagnosis with varying definitions of oligohydramnios in the literature. Potential etiologies for oligohydramnios are diverse and this finding is less specific than Doppler velocities in predicting adverse perinatal outcomes [24]. In the absence of evidence of rupture of membranes or fetal anomalies contributing to the pathology, oligohydramnios is often interpreted as a marker for placental insufficiency and therefore pathology. Though an estimated fetal weight below the tenth percentile in association with oligohydramnios does not predict adverse outcomes, it does suggest pathologic FGR more so than an isolated finding of a small fetus. Therefore, in an attempt to improve the sensitivity of the case definition for FGR oligohydramnios has been included in the definition realizing it may decrease specificity.

Additional sensitivity in the definition could be achieved by tracking an individual fetus’s growth over time. Serial ultrasound would improve sensitivity for FGR through identification of fetuses failing to reach their growth potential but not falling below the tenth percentile. The improvement in sensitivity would come at a tradeoff for additional ultrasound scans which may be overly cumbersome in the context of a vaccine trial. In the interest of feasibility these criteria were not included in the case definition.

The current case definition balances sensitivity and specificity of the definition of FGR. However great care was taken to consider the feasibility of the definition within the context of a vaccine trial with the potential for study sites with varying access to healthcare resources and technology. Access to basic B-mode ultrasound is requisite for the diagnosis at any level of certainty, with a requirement for basic Doppler capabilities for the highest level of diagnostic certainty. In an attempt to make a Level One diagnosis of FGR accessible, after thoughtful consideration more complex criteria that could improve the sensitivity or specificity of detection of disorders of fetal growth potential were excluded.

#### 1.3.4 Timing post immunization

Specific time frames for onset of symptoms following immunization are not included in the definition of FGR. There is a paucity of data about relationship between immunization and FGR with no evidence on which to base recommendations on a temporal relationship. We postulate that a definition designed to be a suitable tool for testing causal relationships requires ascertainment of the outcome (e.g. FGR) independent from the exposure (e.g. immunizations). Therefore, to avoid selection bias, a restrictive time interval from immunization to onset of FGR should not be an integral part of such a definition. Instead, where feasible, details of this interval should be assessed and reported as described in the data collection guidelines.

Further, FGR often occurs outside the controlled setting of a clinical trial or hospital. When possible, a sonographic assessment of fetal weight before vaccination could aid in the evaluation of FGR prior to vaccine administration. In cases where FGR can be identified prior to vaccine administration exclusion of these pregnancies from the trial can be considered. Given the increase in adverse events in pregnancies affected by FGR this strategy may avoid an overestimation of associations between the vaccine and an adverse outcome. In some settings it may be impossible to screen for FGR or obtain a clear timeline of the event, particularly in less developed or rural settings. In order to avoid selecting against such cases, the Brighton Collaboration case definition avoids setting requirements for FGR screening and arbitrary time frames.

### 1.4 Guidelines for data collection, analysis and presentation

As mentioned in the overview paper, the case definition is accompanied by guidelines which are structured according to the steps of conducting a clinical trial, i.e. data collection, analysis and presentation. Neither case definition nor guidelines are intended to guide or establish criteria for management of ill infants, children, or adults. Both were developed to improve data comparability.

### 1.5 Periodic review

Similar to all Brighton Collaboration case definitions and guidelines, review of the definition with its guidelines is planned on a regular basis (i.e. every three to five years) or more often if needed.

## 2 Case definition of fetal growth restriction

### 2.1 For all levels of diagnostic certainty

Fetal growth restriction is a sonographic finding characterized by:

**Level 1 of diagnostic certainty**

***Level 1a***

Level 1^∗^ evidence of pregnancy dating [67].
AND
Estimated fetal weight below 3% using locally-accepted growth curve.
OR
Estimated fetal weight below 10% using locally-accepted growth curve.
  AND
  Absent or reversed end-diastolic flow of the umbilical artery Doppler.
  OR
  Oligohydramnios.^†^

***Level 1b***

Level 1^∗^ evidence of pregnancy dating [67].
AND
Estimated fetal weight below 10%ile using locally-accepted growth curve
AND
Lack of absent or reversed end-diastolic flow of the umbilical artery or oligohydramnios.^†^

**Level 2 of diagnostic certainty**

***Level 2a***

Level 2 evidence of pregnancy dating [67].
AND
Estimated fetal weight below 3% using locally-accepted growth curve
OR
Estimated fetal below 10% using locally-accepted growth curve
  AND
  Absent or reversed end-diastolic flow of the umbilical artery Doppler.
  OR
  Oligohydramnios^†^

***Level 2b***

Level 2 evidence of pregnancy dating [67].
AND
Estimated fetal weight below 10%ile using locally-accepted growth curve
AND
No findings of absent or reversed end-diastolic flow of the umbilical artery or oligohydramnios^†^.
OR
Level 1^∗^ evidence of pregnancy dating [67].
AND
Estimated fetal weight below 10% using locally-accepted growth curve with no findings of oligohydramnios^†^ with inability to assess umbilical artery Doppler.

### 2.2 Insufficient evidence

Absence of ultrasound for use in assessment of estimated fetal weight.

^*^Level 1 evidence of pregnancy dating as defined by the Preterm Birth Working Group of the Brighton Collaboration. Level 1 pregnancy dating depends on a confirmatory ultrasound performed ⩽13 6/7 weeks gestation [67].

^†^Oligohydramnios is defined as a decreased amniotic fluid volume as defined by amniotic fluid index less than 8 cm or deepest vertical pocket less than 2 cm in the presence of intact membranes without concern for fetal anomalies contributing to its etiology. When compared to umbilical artery Dopplers, oligohydramnios lacks specificity for pathologic placental underperfusion and lacks association with adverse perinatal outcomes [24]. It is, however, a useful sonographic findings in situations lacking access to Doppler ultrasound and often impacts clinical management of pregnancies with suspected FGR despite its lack of specificity.

## 3 Guidelines for data collection, analysis and presentation of fetal growth restriction

It was the consensus of the Brighton Collaboration *Fetal Growth Restriction Working Group* to recommend the following guidelines to enable meaningful and standardized collection, analysis, and presentation of information about FGR. However, implementation of all guidelines might not be possible in all settings. The availability of information may vary depending upon resources, geographical region, and whether the source of information is a prospective clinical trial, a post-marketing surveillance or epidemiological study, or an individual report of FGR. Also, as explained in more detail in the overview paper in this volume, these guidelines have been developed by this working group for guidance only, and are not to be considered a mandatory requirement for data collection, analysis, or presentation.

### 3.1 Data collection

These guidelines represent a desirable standard for the collection of data on availability following immunization to allow for comparability of data, and are recommended as an addition to data collected for the specific study question and setting. The guidelines are not intended to guide the primary reporting of FGR to a surveillance system or study monitor. Investigators developing a data collection tool based on these data collection guidelines also need to refer to the criteria in the case definition, which are not repeated in these guidelines.

Guidelines 1–42 below have been developed to address data elements for the collection of adverse event information as specified in general drug safety guidelines by the International Conference on Harmonization of Technical Requirements for Registration of Pharmaceuticals for Human Use, and the form for reporting of drug adverse events by the Council for International Organizations of Medical Sciences [68], [69]. These data elements include an identifiable reporter and patient, one or more prior immunizations, and a detailed description of the adverse event, in this case, of FGR following immunization. The additional guidelines have been developed as guidance for the collection of additional information to allow for a more comprehensive understanding of FGR following immunization.

#### 3.1.1 Source of information/reporter

For all cases and/or all study participants, as appropriate, the following information should be recorded:

(1) Date of report.
(2) Name and contact information of person reporting^3^ and/or diagnosing the FGR as specified by country-specific data protection law.
(3) Name and contact information of the investigator responsible for the subject, as applicable.
(4) Relation to the patient (e.g., immunizer [clinician, nurse], family member [indicate relationship], other).

#### 3.1.2 Vaccinee/control

##### 3.1.2.1 Demographics

For all cases and/or all study participants, as appropriate, the following information should be recorded:

(5) Case/study participant identifiers (e.g. first name initial followed by last name initial) or code (or in accordance with country-specific data protection laws).
(6) Date of birth, age, and sex.
(7) For infants: Gestational age and birth weight.

##### 3.1.2.2 Clinical and immunization history

For all cases and/or all study participants, as appropriate, the following information should be recorded:

(8) Past medical history, including hospitalizations, underlying diseases/disorders with careful identification as to their presence in the mother or fetus, pre-immunization signs and symptoms including identification of indicators for, or the absence of, a history of allergy to vaccines, vaccine components or medications; food allergy; allergic rhinitis; eczema; asthma.
(9) Any medication history (other than treatment for the event described) prior to, during, and after immunization including prescription and non-prescription medication as well as medication or treatment with long half-life or long term effect. (e.g. immunoglobulins, blood transfusion and immunosuppressants).
(10) Immunization history (i.e. previous immunizations and any adverse event following immunization (AEFI)), in particular occurrence of FGR after a previous immunization.

#### 3.1.3 Details of the immunization

For all cases and/or all study participants, as appropriate, the following information should be recorded:

(11) Date and time of immunization(s).
(12) Description of vaccine(s) (name of vaccine, manufacturer, lot number, dose (e.g. 0.25 mL, 0.5 mL, etc.), diluent name, and number of dose if part of a series of immunizations against the same disease).
(13) The anatomical sites (including left or right side) of all immunizations (e.g. vaccine A in proximal left lateral thigh, vaccine B in left deltoid).
(14) Route and method of administration (e.g. intramuscular, intradermal, subcutaneous, and needle-free (including type and size), other injection devices).
(15) Needle length and gauge.

#### 3.1.4 The adverse event

(16) For all cases at any level of diagnostic certainty and for reported events with insufficient evidence, the criteria fulfilled to meet the case definition should be recorded.

Specifically document:

(17) Sonographic findings of FGR and if there was medical confirmation of the event (i.e. patient seen or images reviewed by physician).
(18) Date/time of onset,^4^ first observation^5^ and diagnosis,^6^ end of episode^7^ and final outcome.^8^
(19) Concurrent signs, symptoms, and diseases.^9^
(20) Measurement/testing
  • Values and units of routinely measured parameters (e.g. measurements, amniotic fluid volume, umbilical artery Doppler studies, additional Doppler studies) – in particular those indicating the severity of the event;
  • Method of measurement (e.g. type of ultrasound, particular growth curve or references used);
  • Results of laboratory examinations, surgical and/or pathological findings and diagnoses if present.
(21) Follow up given for FGR, especially follow up monitoring (e.g. repeat ultrasounds or other assessments of fetal well-being) and delivery planning.
(22) Outcome^8^ at last observation.
(23) Exposures other than the immunization 24 h before and after immunization (e.g. food, environmental) considered potentially relevant to the reported event.

#### 3.1.5 Miscellaneous/general

(24) The duration of surveillance for FGR should be predefined based on
  • Biologic characteristics of the vaccine e.g. live attenuated versus inactivated component vaccines;
  • Biologic characteristics of the vaccine-targeted disease;
  • Biologic characteristics of FGR including patterns identified in previous trials (e.g. early-phase trials); and
  • Biologic characteristics of the vaccinee (e.g. nutrition, underlying disease like immunodepressing illness).
(25) The duration of follow-up reported during the surveillance period should be predefined likewise. It should aim to continue to resolution of the event.
(26) Methods of data collection should be consistent within and between study groups, if applicable.
(27) Follow-up of cases should attempt to verify and complete the information collected as outlined in data collection guidelines 1 to 23.
(28) Investigators of patients with FGR should provide guidance to reporters to optimize the quality and completeness of information provided.
(29) Reports of FGR should be collected throughout the study period regardless of the time elapsed between immunization and the adverse event. If this is not feasible due to the study design, the study periods during which safety data are being collected should be clearly defined.

### 3.2 Data analysis

The following guidelines represent a desirable standard for analysis of data on FGR to allow for comparability of data, and are recommended as an addition to data analyzed for the specific study question and setting.

(30) Reported events should be classified in one of the following five categories including the three levels of diagnostic certainty. Events that meet the case definition should be classified according to the levels of diagnostic certainty as specified in the case definition. Events that do not meet the case definition should be classified in the additional categories for analysis.
**Event classification in 4 categories**^10^
**Event meets case definition**
  (1) Level 1: Criteria as specified in the Fetal Growth Restriction case definition
  (2) Level 2: Criteria as specified in the Fetal Growth Restriction case definition
**Event does not meet case definition**
***Additional categories for analysis***
  (1) Reported FGR with insufficient evidence to meet the case definition^11^
  (2) Not a case of FGR
(31) The interval between immunization and reported FGR could be defined as the date/time of immunization to the date/time of onset^4^ of the first symptoms and/or signs consistent with the definition. If few cases are reported, the concrete time course could be analyzed for each; for a large number of cases, data can be analyzed in the following increments:

**Subjects with Fetal Growth Restriction by Interval to Presentation**

**Table:** 

Interval^∗^	Number
0 – <2 weeks after immunization	
2 – <4 weeks after immunization	
4 – <8 weeks after immunization	
8 – <12 weeks after immunization	
Week increments thereafter	
**TOTAL**

(32) The duration of a possible FGR could be analyzed as the interval between the date/time of onset^3^ of the first sonographic findings consistent with the definition and the end of diagnosis^7^ and/or final outcome.^8^ Whatever start and ending are used, they should be used consistently within and across study groups.
(33) If more than one measurement of a particular criterion is taken and recorded, the value corresponding to the greatest magnitude of the adverse experience could be used as the basis for analysis. Analysis may also include other characteristics like qualitative patterns of criteria defining the event.
(34) The distribution of data (as numerator and denominator data) could be analyzed in predefined increments (e.g. measured values, times), where applicable. Increments specified above should be used. When only a small number of cases are presented, the respective values or time course can be presented individually.
(35) Data on FGR obtained from subjects receiving a vaccine should be compared with those obtained from an appropriately selected and documented control group(s) to assess background rates of FGR in non-exposed populations, and should be analyzed by study arm and dose where possible, e.g. in prospective clinical trials [47].

### 3.3 Data presentation

These guidelines represent a desirable standard for the presentation and publication of data on FGR following immunization to allow for comparability of data, and are recommended as an addition to data presented for the specific study question and setting. Additionally, it is recommended to refer to existing general guidelines for the presentation and publication of randomized controlled trials, systematic reviews, and meta-analyses of observational studies in epidemiology (e.g. statements of Consolidated Standards of Reporting Trials (CONSORT), of Improving the quality of reports of meta-analyses of randomized controlled trials (QUORUM), and of meta-analysis Of Observational Studies in Epidemiology (MOOSE), respectively) [70], [71], [72].

(36) All reported events of FGR should be presented according to the categories listed in guideline 30.
(37) Data on possible FGR events should be presented in accordance with data collection guidelines 1–23 and data analysis guidelines 30–35.
(38) Terms to describe FGR such as “low-grade”, “mild”, “moderate”, “high”, “severe” “significant”, “early onset”, “late onset”, “asymmetric”, “symmetric” are highly subjective, prone to wide interpretation, and should be avoided, unless clearly defined.
(39) Data should be presented with numerator and denominator (n/N) (and not only in percentages), if available.

Although immunization safety surveillance systems denominator data are usually not readily available, attempts should be made to identify approximate denominators. The source of the denominator data should be reported and calculations of estimates be described (e.g. manufacturer data like total doses distributed, reporting through Ministry of Health, coverage/population based data, etc.).

(40) The incidence of cases in the study population should be presented and clearly identified as such in the text.
(41) If the distribution of data is skewed, median and range are usually the more appropriate statistical descriptors than a mean. However, the mean and standard deviation should also be provided.
(42) Any publication of data on FGR should include a detailed description of the methods used for data collection and analysis as possible. It is essential to specify:
(43) The study design;
  • The method of pregnancy dating;
  • The method, frequency and duration of monitoring for FGR;
  • The trial profile, indicating participant flow during a study including drop-outs and withdrawals to indicate the size and nature of the respective groups under investigation;
  • The type of surveillance (e.g. passive or active surveillance);
  • The characteristics of the surveillance system (e.g. population served, mode of report solicitation);
  • The search strategy in surveillance databases;
  • Comparison group(s), if used for analysis;
  • The validated instrument of data collection (e.g. standardized questionnaire, diary card, report form);
  • Whether the day of immunization was considered “day one” or “day zero” in the analysis;
  • Whether the date of onset^4^ and/or the date of first observation^5^ and/or the date of diagnosis^6^ was used for analysis; and^12^
  • Use of this case definition for FGR, in the abstract or methods section of a publication.^13^

## Disclaimer

The findings, opinions and assertions contained in this consensus document are those of the individual scientific professional members of the working group. They do not necessarily represent the official positions of each participant’s organization (e.g., government, university, or corporation). Specifically, the findings and conclusions in this paper are those of the authors and do not necessarily represent the views of their respective institutions.

## Footnotes

3 If the reporting center is different from the vaccinating center, appropriate and timely communication of the adverse event should occur.

4 The date and/or time of onset is defined as the time post immunization, when the first sign or symptom indicative for FGR occurred. This may only be possible to determine in retrospect.

5 The date and/or time of first observation of the first sign or symptom indicative for FGR can be used if date/time of onset is not known.

6 The date of diagnosis of an episode is the day post immunization when the event met the case definition at any level.

7 The end of an episode is defined as the time the event no longer meets the case definition at the lowest level of the definition.

8 E.g. recovery to pre-immunization health status, spontaneous resolution, therapeutic intervention, persistence of the event, sequelae, death.

9 An AEFI is defined as serious by international standards if it meets one or more of the following criteria: (1) it results in death, (2) is life-threatening, (3) it requires inpatient hospitalization or results in prolongation of existing hospitalization, (4) results in persistent or significant disability/incapacity, (5) is a congenital anomaly/birth defect, (6) is a medically important event or reaction.

10 To determine the appropriate category, the user should first establish, whether a reported event meets the criteria for the lowest applicable level of diagnostic certainty, e.g. Level Two B. If the lowest applicable level of diagnostic certainty of the definition is met, and there is evidence that the criteria of the next higher level of diagnostic certainty are met, the event should be classified in the next category. This approach should be continued until the highest level of diagnostic certainty for a given event could be determined. If the lowest level of the case definition is not met, it should be ruled out that any of the higher levels of diagnostic certainty are met and the event should be classified in additional categories three or four.

11 If the evidence available for an event is insufficient because information is missing, such an event should be categorized as “Reported FGR with insufficient evidence to meet the case definition”.

12 An event does not meet the case definition if investigation reveals a negative finding of a necessary criterion (necessary condition) for diagnosis. Such an event should be rejected and classified as “Not a case of FGR”.

13 Use of this document should preferably be referenced by referring to the respective link on the Brighton Collaboration website (http://www.brightoncollaboration.org).
